# Tear fluid CGRP rises during glyceryl trinitrate-induced headache in migraine patients

**DOI:** 10.1186/s10194-025-02141-w

**Published:** 2025-11-14

**Authors:** Katharina Kamm, Marie-Christine Khorsandian, Andreas Straube, Ruth Ruscheweyh

**Affiliations:** 1 Department of Neurology, University Hospital, LMU Munich, Munich, Germany; 2 Department of Psychiatry, Psychotherapy and Psychosomatics, Psychiatric Hospital, University of Zurich, Zurich, Switzerland; 3 Department of Neurology, University Hospital Zurich, University of Zurich, Zurich, Switzerland

**Keywords:** Migraine, CGRP, GTN, Headache, Tear fluid

## Abstract

**Background:**

Calcitonin gene-related peptide (CGRP) plays an important role in migraine pathophysiology and is a marker of trigeminal activity. We investigated tear fluid CGRP levels interictally and ictally using the glyceryl trinitrate (GTN) experimental migraine model.

**Methods:**

Study participants were assessed interictally and during an experimental headache using GTN. Interictal tear fluid CGRP levels were assessed in episodic migraine (EM) patients and healthy controls (HC). At the day of experimental headache, tear fluid was sampled in EM patients before and after intravenous GTN application. Participants were free to use their usual abortive medication if needed. Tear fluid CGRP levels were analyzed using two different ELISAs.

**Results:**

26 EM patients and 20 healthy controls were examined interictally. Interictal tear fluid CGRP was significantly higher in EM patients compared to HCs (Abbexa® CGRP ELISA: interictal EM patients: 2.78 ± 1.99 ng/ml, HC: 1.35 ± 0.78 ng/ml, U = −2.969, *p* = 0.003). 17 EM patients were analyzed after intravenous GTN. Tear fluid CGRP levels were significantly elevated at maximum headache compared to baseline (Abbexa® CGRP ELISA: 3.84 ± 1.96 ng/ml vs. 2.78 ± 2.38 ng/ml, *p* = 0.025; Cusabio® CGRP ELISA: 1.43 ± 0.96 ng/ml vs. 0.75 ± 0.77 ng/ml, *p *< 0.001). Seven patients developed a headache with an intensity ≥ 5 on the numerical rating scale (NRS) and showed a significantly higher rise in tear fluid CGRP compared to patients with a headache < 5 on the NRS (Abbexa® CGRP ELISA: + 2.11 ± 2.14 ng/ml vs. + 0.32 ± 1.33 ng/ml, *p* = 0.033; Cusabio® CGRP ELISA: + 1.45 ± 0.81 ng/ml vs. + 0.09 ± 0.53 ng/ml; *p* < 0.001). Tear fluid CGRP levels were significantly more reduced after intake of abortive medication than after spontaneous headache improvement (Abbexa® CGRP ELISA: −0.82 ± 1.26 ng/ml vs. 1.24 ± 2.06 ng/ml, *p* = 0.014; Cusabio® CGRP ELISA: −0.66 ± 1.27 ng/ml vs. −0.12 ± 0.24 ng/ml, *p* = 0.033).

**Conclusion:**

The present results demonstrate validity of tear fluid CGRP assessment during experimental migraine, detecting a rise of tear fluid CGRP levels during GTN-induced headache. The intake of abortive medication reduced tear fluid CGRP levels significantly more than spontaneous headache improvement.

**Graphical abstract:**

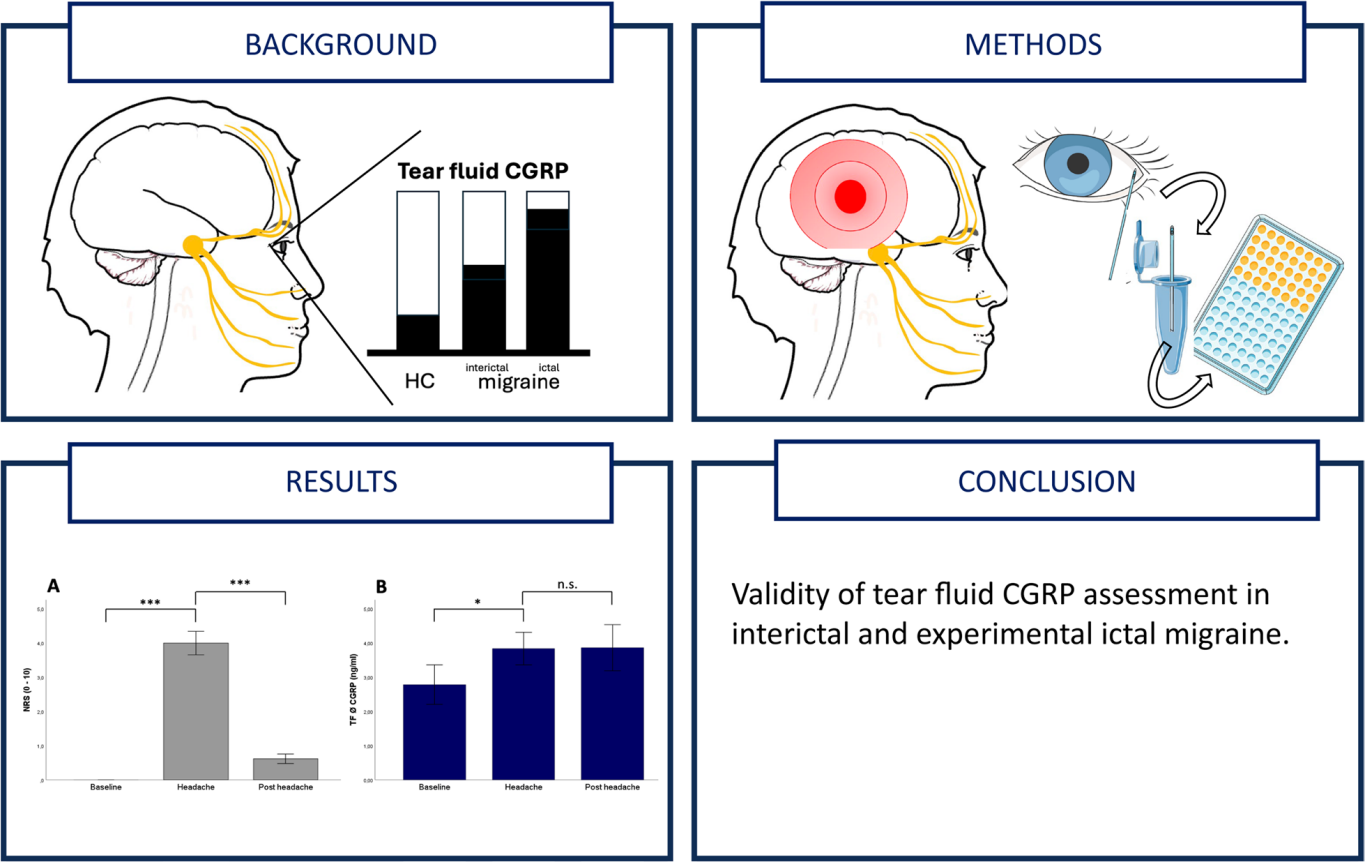

## Key findings

- Tear fluid CGRP rises during headache, even more so if headache is intense. This rise can be detected by both used ELISAs (Abbexa® and Cusabio® CGRP ELISA)
- Tear fluid CGRP decreases after successful treatment with an abortive medication.
- Detection of CGRP in tear fluid is feasible and valid interictally and during acute headache.

## Introduction

Migraine is a common neurological disorder affecting 15.3% of the global population [1]. The International Classification of Headache Disorders, 3rd version (ICHD-3) defines this primary headache disorder by recurring, often unilateral, pulsating, moderate to severe headache attacks accompanied by photo- or phonophobia, nausea and/or vomiting [2].

The importance of the trigemino-vascular system and the neuropeptide calcitonin gene-related peptide (CGRP) in migraine pathophysiology has been shown in numerous studies [3, 4]. CGRP is expressed in cells of C-type sensory pain fibers in the trigeminal ganglion. The neuropeptide is released from these cells peripherally and centrally. Peripherally, the trigeminal nerve innervates cerebral blood vessels, the dural vasculature and the dura mater. The dural release of CGRP and other neuropeptides upon stimulation leads to neurogenic inflammation which is thought to induce migraine attacks [4, 5]. Besides the innervation of the meninges mainly by the first branch of the trigeminal nerve (V1), the eye and its associated structures are also innervated by the trigeminal nerve [6]. Centrally, CGRP-containing fibers project to the spinal trigeminal nucleus, the dorsal horns of the spinal cord (C1 and C2), as well as to other structures in the CNS involved in pain processing [4, 5].

Elevated CGRP levels were first shown in blood of the external jugular vein during a migraine attack [7]. Later, elevated CGRP levels in peripheral blood have been shown in interictal and ictal migraine patients, however the peripheral detection of CGRP is still debated [4, 8]. Detection of CGRP in peripheral blood showed inconsistent findings, probably due to fast degradation and high dilution of the peptide in systemic circulation [8]. Due to the direct trigeminal innervation of salivary glands and the eye, these compartments are alternatives for the detection of CGRP and both methods have been tested previously [9–13]. In a former study, we detected elevated interictal and ictal tear fluid CGRP levels in migraine patients compared to healthy controls (HC) [12].

To further investigate the role of CGRP during the course of a migraine attack, the current study used glyceryl trinitrate (GTN) as a human experimental migraine model [14]. An experimental migraine induced by GTN was first described in 1987. It was shown that migraine patients more often developed a headache after sublingual GTN application than healthy controls [15]. In addition to an immediate headache during GTN application also observed in healthy controls, migraine patients often developed a more severe, delayed headache (> 1 h after GTN administration) reminiscent of their usual migraine attacks. Subsequently, GTN has been validated as an experimental migraine model and applied in a multitude of experimental migraine studies [16]. Elevated plasma CGRP levels in migraine-like headache after GTN and normalization after intake of abortive medication has been shown [17, 18].

The aim of the present study was to investigate interictal and ictal tear fluid CGRP levels in episodic migraine (EM) patients. To investigate ictal tear fluid CGRP levels the GTN experimental migraine model was used. It was hypothesized that ictal tear fluid CGRP levels are elevated compared to interictal tear fluid CGRP levels.

## Methods

### Participants

Participants were recruited at our outpatient headache center and by advertisements at the University Hospital of the Ludwig-Maximilians-University Munich. The study was conducted in accordance with the Declaration of Helsinki and was approved by the LMU ethics committee (18–827). All participants gave written informed consent.

Inclusion criteria were as follows: age between 18 and 65 years, participants with episodic migraine with or without aura (with 1–14 headache days per month) according to the ICHD-3 criteria [2] or healthy controls. Exclusion criteria were: the presence of other primary headaches in migraine patients or any primary headaches in healthy controls, wearing contact lenses on the days of study participation, pre-existing neurological, ophthalmological or severe medical or psychiatric conditions. Breast-feeding or pregnant women were excluded. Further, subjects with known arterial hypertension or a blood pressure of  > 140/90 mmHg on the study day were excluded. To ensure interictal status, participants were only included if they were headache- and abortive medication-free 48 h prior to and after study participation and had no contraindications to GTN application like a known allergy or severe hypotension. A stable preventive medication for migraine was allowed.

### Study procedure

The study was conducted between 08/2019 and 10/2020 at the outpatient headache center at LMU university hospital; the study comprises two investigations, an interictal and ictal assessment of tear fluid CGRP.

The interictal assessment examined CGRP levels in tear fluid in interictal episodic migraine patients compared to healthy controls. EM patients and HCs were thoroughly interviewed regarding their migraine history (if applicable), other medical conditions and regular intake of medication. Blood pressure was measured and subsequently, tear fluid samples from both eyes were collected as described below.

For investigation of ictal CGRP levels, an experimental headache was induced using GTN. On the ictal study day, participants presented to our outpatient headache center between 8 and 9 am and baseline tear fluid was collected. Then, participants received GTN (0.5 µg/kg BW/min) intravenously over 20 min using an infusion pump and vital signs were recorded every 5 min. Subsequently, tear fluid was collected regularly every 60 min and patients filled a headache questionnaire before every sampling, rating headache intensity on a numerical rating scale (NRS) from 0 to 10 and the presence of migraine symptoms. Patients were allowed to use their regular abortive medication (AM) as needed. If so, an additional sample was collected before intake of the medication. On average, 7 ± 1 tear fluid samples were collected per patient. One to two hours after spontaneous or drug-related substantial improvement of headache, patients were discharged, in total study participants were observed for 8–9 h after GTN infusion.

**Fig. 1:**
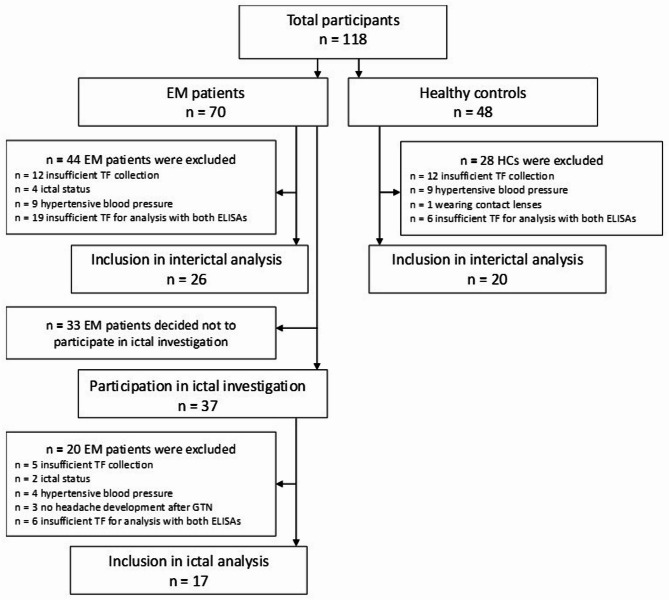
Participant disposition. 70 EM patients and 48 HCs were enrolled in interictal assessment, of these 44 EM patients were excluded due to insufficient tear fluid collection (n = 12) or not meeting inclusion criteria (current migraine attack or intake of abortive medication (n = 4), hypertensive blood pressure (n = 9)) or due to insufficient tear fluid sample for analysis with two different ELISAs (n = 19). 28 HCs were excluded from analysis due to insufficient tear fluid collection (n = 12), not meeting inclusion criteria (hypertensive blood pressure (n = 9), wearing contact lenses (n = 1) or insufficient tear fluid amount for the analysis using two different ELISAs (n = 6). 37 EM patients participated the ictal assessment, of these 20 patients were excluded because of insufficient amount of tear fluid (n = 5), hypertensive blood pressure (n = 4), and ictal status (n = 2). 3 further patients were excluded from the analysis because they did not develop a headache within 7 h after GTN infusion, precluding group comparison at the time of maximum headache. Further, 6 patients were excluded due to insufficient sample material to analyze the probe using both ELISAs

Baseline CGRP levels (before GTN infusion, ‘baseline’) were compared to CGRP levels at maximum headache intensity (‘headache’) and to those after headache improvement (at the time of lowest headache intensity during the 1–2 h after abortive medication intake or spontaneous improvement, ‘post headache’). Further, a group comparison based on headache intensity and intake of abortive medication (AM) was performed. 70 episodic migraine patients and 48 healthy controls participated interictal assessment, of these 26 EM patients and 20 HCs were included in analysis. Of 37 EM patients attending the ictal investigation, 17 migraine patients were included in analysis (see Fig. 1 for participant disposition).

Tear fluid and CGRP measurement was performed as previously described [12, 13]. Briefly, tear fluid was collected from both eyes separately after participants rested supine for 5 min. A plastic capillary (ref. no. 100012, Sanguis, Nümbrecht, Germany) was dipped into tear fluid at the lateral canthus and removed after complete filling or a maximum sampling time of 1 min. Much care was taken not to irritate the eye during tear fluid collection and the procedure was stopped if the eye showed signs of excessive tearing in order to prevent dilution of samples. Tear fluid obtained was immediately immersed in a 1.5 ml tube containing 500µl of tissue protein extractor solution (TPER; Pierce Rockford, IL), centrifuged for 5 min at 4000g and stored at -80°C.

Tear fluid CGRP levels were determined using the commercial Cusabio® and Abbexa® CGRP ELISA kit (Cusabio®, Wuhan, China; detection range: 1.56—100 pg/ml, minimal detectable dose: 0.39 pg/ml, intra-assay precision: < 8%, inter-assay precision: < 10%; Abbexa®, Cambridge, United Kingdom; detection range: 3.13–200 pg/ml, minimal detectable dose: 1.88 pg/ml, intra-assay precision: < 8%, inter-assay precision: < 10%), following manufacturer’s instructions. Duplicate measurements with each ELISA were performed for each sample. Absorbance values were read using a BioRad spectrometer (BioRad Laboratories Inc., USA) and CGRP concentrations were determined from calibration curves using a 4PL fitting. The final CGRP concentration of each sample was calculated as the average of the two measurements. Interictal tear fluid CGRP levels did not differ between the right and left eye of migraine patients (Abbexa® CGRP ELISA: right eye: 2.89 ± 2.17 ng/ml, left eye: 2.66 ± 2.06 ng/ml; z = −1.206, *p* = 0.228, n = 26. Cusabio® CGRP ELISA: right eye: 1.33 ± 2.00, left eye: 0.96 ± 1.31; z = −0.698 m *p* = 0.485) and controls (Abbexa® CGRP ELISA: right eye: 1.59 ± 0.84 ng/ml; left eye: 1.32 ± 0.61 ng/ml; z = −1.755, *p* = 0.079. Cusabio® CGRP ELISA: right eye: 1.22 ± 1.53 ng/ml, left eye: 0.74 ± 0.74 ± 0.47 ng/ml; z = −1.083, *p* = 0.279).

### Statistics

Data is presented as mean ± standard deviation unless stated otherwise. According to the Shapiro–Wilk-Test some data was non-normally distributed, so median and interquartile ranges (IQR) are additionally displayed in the text, if appropriate. Further, non-parametric tests were used. For comparison of age, gender, monthly headache and migraine days and headache intensity, chi-square test or Mann–Whitney U test were used. For comparison of headache intensity and CGRP levels over different points in time, Friedman test was used followed by posthoc Dunn-Bonferroni tests if appropriate. For comparison of headache intensity or CGRP levels between different groups (NRS ≥ 5 vs. NRS < 5, intake of abortive medication vs. no intake), Mann–Whitney U test was used. Statistical analysis was performed with SPSS 29 (IBM, Corp., Armonk, NY, USA). Significance was accepted at *p* < 0.05 (two-tailed).

## Results

### Interictal tear fluid CGRP levels were elevated in migraine patients compared to healthy controls

**Fig. 2:**
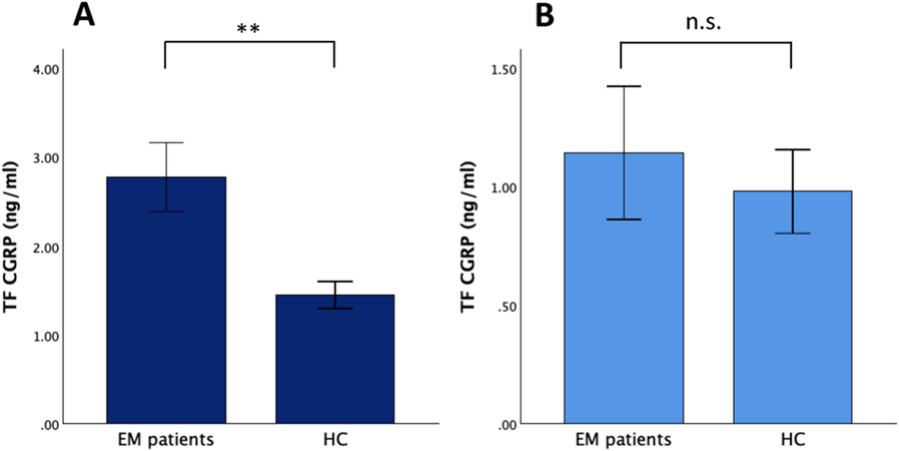
Tear fluid CGRP levels in episodic migraine patients and healthy controls. (**A**) Tear fluid (TF) CGRP levels were significantly higher in 26 interictal EM patients (2.78 ± 1.99 ng/ml) compared to 20 healthy controls (1.35 ± 0.78 ng/ml, U = 71.000, *p *= 0.007) in the Abbexa® CGRP ELISA. (**B**) There was no significant difference in TF CGRP levels in EM patients (1.14 ± 1.44 ng/ml) compared to 20 HCs (0.98 ± 0.79 ng/ml; U = 297.000, *p* = 0.412) using the Cusabio® CGRP ELISA. Bars represent standard error, ** p < 0.01, n.s. not significant

Twenty-six migraine patients (f = 21, 24.1 ± 4.3 years, median (IQR) 23 (7) years) and 20 healthy control participants (f = 14, 26.6 ± 5.5 years, median (IQR) 26 (7) years) were included in the analysis (see Table 1 for patient characteristics). Interictal tear fluid CGRP was significantly higher in episodic migraine patients compared to healthy controls using the Abbexa® CGRP ELISA (Abbexa® CGRP ELISA: interictal EM patients: 2.78 ± 1.99 ng/ml, median (IQR) 2.28 (2.11) ng/ml, healthy controls: 1.35 ± 0.78 ng/ml, median (IQR) 1.68 (1.20) ng/ml; U = 71.000, *p* = 0.007, Fig. 2A). With the Cusabio® CGRP ELISA, although migraine patients on average had higher CGRP levels than controls, the difference did not reach significance (Cusabio® CGRP ELISA: interictal EM patients: 1.14 ± 1.44 ng/ml, median (IQR) 0.56 (0.99) ng/ml, healthy controls: 0.98 ± 0.79 ng/ml, median (IQR) 0.76 (0.71) ng/ml; U = 297.000, *p* = 0.412, Fig. 2B).

**Table 1:** Characteristics of the study population. Interictal EM patients were compared to healthy controls

	EM patients	Healthy controls	Statistics
n (% f)	26 (80.8%)	20 (70.0%)	χ^2^(1) = 0.721, *p* = 0.396
Age (in years)	24.1 ± 4.3	26.6 ± 5.5	U = −1.693; *p* = 0.091
Monthly headache days (MHD)	8.0 ± 4.1	–	–
Monthly migraine days (MMD)	3.2 ± 2.4		–
Headache intensity (0–10)	7.8 ± 0.9	–	–
Time since diagnosis (in years)	7.1 ± 5.8	–	–
Prophylactic medication	5 (19.2%)	–	–
*Beta blocker*	3 (11.5%)	–	–
*Amitriptyline*	2 (7.7%)	–	–

The two groups showed no differences in gender distribution or age. EM, episodic migraine

### GTN-induced headache was associated with a rise in tear fluid CGRP

**Fig. 3:**
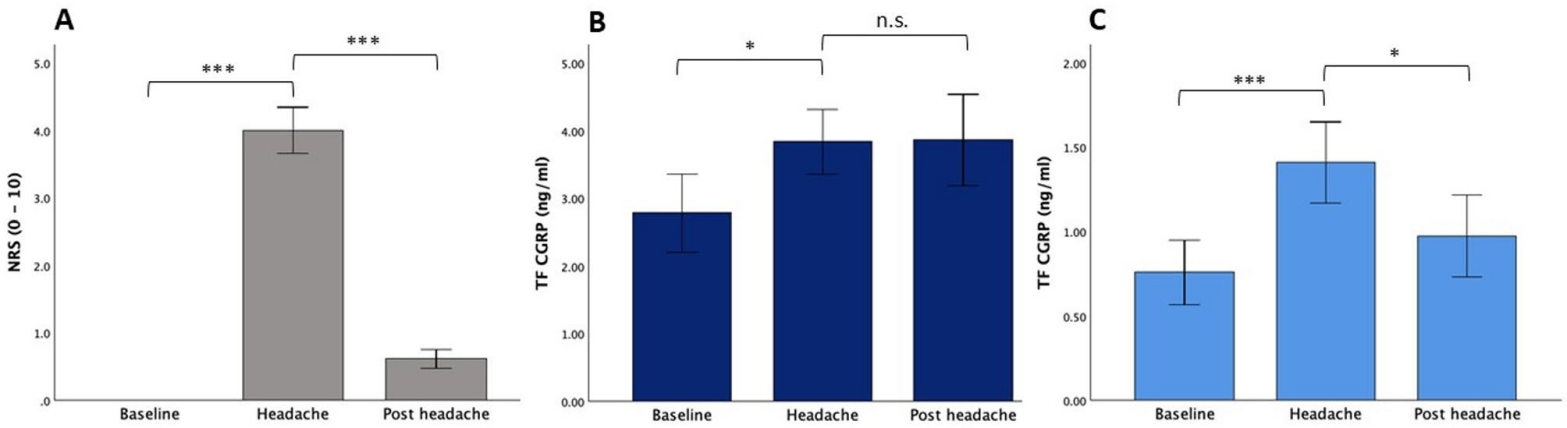
Fig. 3. Headache intensity and TF CGRP levels after GTN infusion. (A) Headache intensity. Before GTN infusion, patients were headache-free (baseline). After GTN application, participants rated their headache intensity regularly. At the timepoint of most intense headache intensity (headache: NRS: 4.0 ± 1.4), tear fluid was collected and participants could take their regular abortive medication. 1 - 2 hours after intake of abortive medication or spontaneous improvement of headache (post headache), tear fluid was collected again. (B) Tear fluid CGRP levels were significantly different over the course of GTN induced headache. Tear fluid CGRP levels were significantly higher during ‘headache’ compared to ‘baseline’ using the Abbexa® CGRP ELISA (base-line: 2.78 ± 2.38 ng/ml, headache: 3.84 ± 1.96 ng/ml, post headache: 3.86 ± 2.79; p = 0.025). (C) Tear fluid CGRP levels were also significantly higher during ‘headache’ compared to ‘baseline’ using the Cusabio® CGRP ELISA (baseline: 0.75 ± 0.77 ng/ml, headache: 1.43 ± 0.96 ng/ml, post headache: 1.00 ± 0.97; p < 0.001). After headache improvement (post headache) tear fluid CGRP levels were significantly reduced compared to ‘headache’ phase (p = 0.040). Bars represent standard error. *** p < 0.001, * p < 0.05, n.s. not significant

Seventeen episodic migraine patients were included in this analysis (see Table 2 for patient characteristics). The effect of GTN infusion on headache intensity ratings and CGRP levels in tear fluid are shown in Fig. 3.

**Table 2:** Characteristics of the study population receiving GTN. NRS ≥ 5, patients who developed an intense headache in response to GTN; NRS < 5, patients who developed at the most a moderate headache in response to GTN (see Methods)

	Total sample	NRS ≥ 5	NRS < 5	
n (% f)	17 (82.4)	7 (71.4)	10 (90.0)	χ^2^(1) = 0.977, *p* = 0.323
Age (in years)	27.1 ± 7.5	28.3 ± 10.8	26.2 ± 4.4	U = 37.000, *p* = 0.887
Monthly headache days (MHD)	7.2 ± 4.0	9.6 ± 4.3	5.5 ± 3.0	U = 14.500, *p* = 0.043
Monthly migraine days (MMD)	3.3 ± 2.7	5.0 ± 3.3	2.1 ± 1.5	U = 15.000, *p* = 0.055
Headache intensity (0–10)	7.9 ± 0.9	8.2 ± 1.1	7.8 ± 0.9	U = 27.000, *p* = 0.475
Time since diagnosis (in years)	6.9 ± 6.2	5.8 ± 4.6	7.6 ± 7.2	U = 22.500, *p* = 0.724
Prophylactic medication	3 (16.7%)	0 (0%)	3 (30.0%)	χ^2^(1) = 2.550, *p* = 0.110
*Beta-blocker*	3 (16.7%)	0 (0%)	3 (30.0%)	–

Statistical group comparisons were performed with χ^2^ test or Mann Whitney U test as appropriate

During GTN infusion, all patients reported the occurrence of mild headache (not shown). After the end of the infusion, patients developed a delayed headache with an average maximum intensity of 4.0 ± 1.4 (range 2 to 6) on the NRS, which was reached at 269 ± 89 min after GTN infusion (‘headache’). Headache improved spontaneously or after use of abortive medication to 0.6 ± 0.6 on the NRS at 395 ± 67 min after GTN (‘post headache’). NRS differences between ‘baseline’ and ‘headache’ and between ‘headache’ and ‘post headache’ were significant (Friedman χ^2^ [2] = 31.705, *p* < 0.001, n = 17; post-hoc Dunn-Bonferroni test ‘baseline’ vs. ‘headache’ z = −1.794, *p* < 0.001; ‘headache’ vs. ‘post headache’ z = 1.206, *p* < 0.001; ‘baseline’ vs. ‘post headache’ z = −0.588, *p* = 0.086, Fig. 3A).

There was a significant rise in tear fluid CGRP from ‘baseline’ to ‘headache’ detected by both ELISAs (Fig. 3B and C). Only the Cusabio® CGRP ELISA detected a significant fall from ‘headache’ to ‘post headache’ (Abbexa® CGRP ELISA: baseline: 2.78 ± 2.38 ng/ml, median (IQR) 2.31 (2.44) ng/ml, headache: 3.84 ± 1.96 ng/ml, median (IQR) 3.32 (2.64) ng/ml, post headache: 3.86 ± 2.79 ng/ml, median (IQR) 3.72 (3.05) ng/ml; Friedman χ^2^ [2]= 7. 412, *p* = 0.025, n = 17; post-hoc Dunn-Bonferroni test: baseline vs. headache z = −0.882, *p* = 0.010; headache vs. post headache z = 0.176, *p* = 0.607; baseline vs. post headache z = −0.706, *p* = 0.040, Fig. 3B. Cusabio® CGRP ELISA: baseline: 0.75 ± 0.77 ng/ml, median (IQR) 0.43 (0.90) ng/ml, headache: 1.43 ± 0.96 ng/ml, median (IQR) 1.18 (1.75) ng/ml, post headache: 1.00 ± 0.97, median (IQR) 0.69 (1.00) ng/ml; Friedman χ^2^[2]= 16.941, *p* < 0.001, n = 17; post-hoc Dunn-Bonferroni test: baseline vs. headache z = -1.412, *p* < 0.001; headache vs. post headache z = 0.706, *p* = 0.040, baseline vs. post headache: z = -0.706, *p* = 0.040, Fig. 3C).

### Higher headache intensity was associated with a larger rise in tear fluid CGRP

**Fig. 4:**
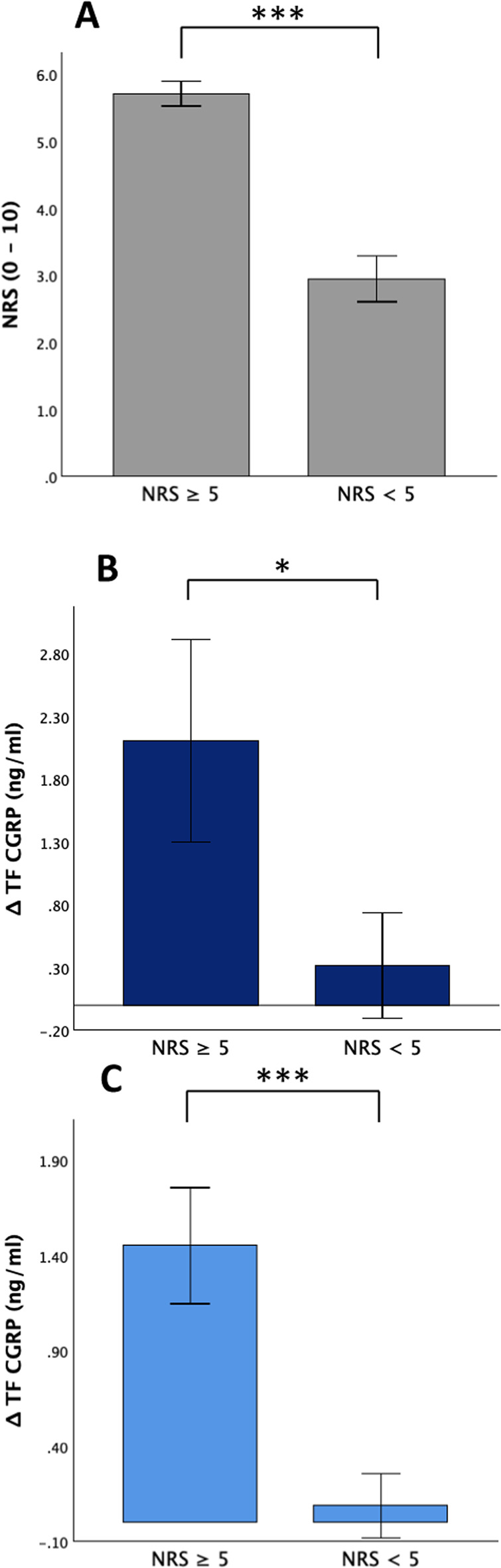
Higher tear fluid CGRP rise in patients with a headache intensity ≥ 5 on the NRS compared to patients with a less intense headache. Changes in NRS and tear fluid CGRP levels from baseline to the time of maximum headache are shown. (**A**) Seven patients stated a significant more intense headache. (**B**, **C**) Tear fluid CGRP levels rose significantly more in patients with an intense headache compared to patients with a moderate headache intensity using the Abbexa® ELISA (*p* = 0.033, B) or the Cusabio® ELISA (*p* < 0.001, C). Bars represent standard error. *** p < 0.001, * p < 0.05

After GTN application, seven migraine patients developed a headache with an intensity ≥ 5 on the NRS (5.7 ± 0.5) which was significantly different from the remaining patients (NRS 3.0 ± 1.1; Mann–Whitney-U-Test: U = 0.000, *p* < 0.001, Fig. 4A). There was no significant difference in ‘baseline’ tear fluid CGRP levels between the two groups (Abbexa® CGRP ELISA: NRS ≥ 5: 1.86 ± 1.30 ng/ml, median (IQR) 1.87 (2.02) ng/ml; NRS < 5: 3.43 ± 2.80 ng/ml, median (IQR) 2.34 (3.37) ng/ml; Mann–Whitney-U-Test: U = 48.000, *p* = 0.230; Cusabio® CGRP ELISA: NRS ≥ 5: 0.73 ± 0.51 ng/ml, median (IQR) 0.53 (0.90) ng/ml; NRS < 5: 0.78 ± 0.97 ng/ml, median (IQR) 0.42 (1.00) ng/ml; Mann–Whitney-U-Test: U = 30.000, *p* = 0.669); however, the NRS ≥ 5 group showed a significantly higher rise of tear fluid CGRP from ‘baseline’ to ‘headache’ compared to NRS < 5 group using both ELISAs (Abbexa® CGRP ELISA: + 2.11 ± 2.14 ng/ml, median (IQR) + 1.94 (1.86) ng/ml vs. + 0.32 ± 1.33 ng/ml, median (IQR) + 0.56 (1.44) ng/ml; Mann–Whitney-U-Test: U = 13.000, *p* = 0.033, Fig. 4B; Cusabio® CGRP ELISA: + 1.45 ± 0.81 ng/ml, median (IQR) + 1.23 (0.84) ng/ml vs. + 0.17 ± 0.29 ng/ml; Mann–Whitney-U-Test: U = 1.000, *p* < 0.001, Fig. 4C).

### Use of attack abortive medication was associated with a larger reduction in tear fluid CGRP

**Fig. 5:**
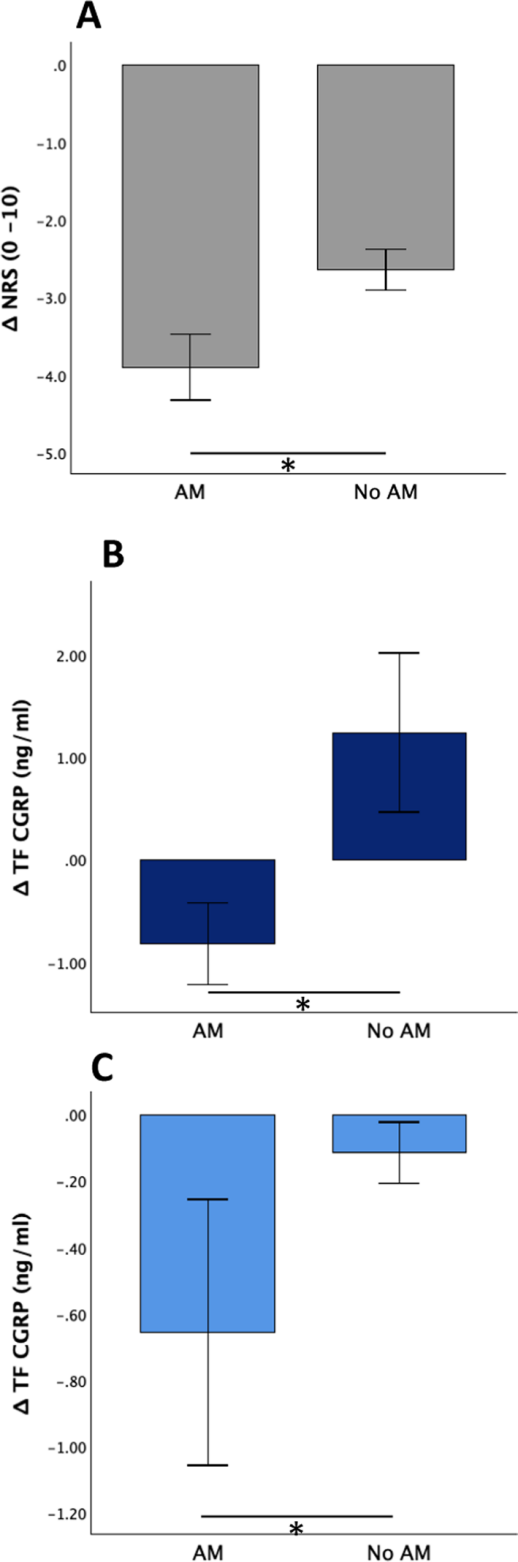
Influence of abortive medication on headache intensity and tear fluid CGRP levels. Changes in NRS and tear fluid CGRP levels from the time of maximum headache to the ‘post headache’ phase are shown. All included patients refrained from taking acute medication 48 h prior to GTN application. Patients who developed a headache after GTN application, were allowed to take their regular abortive medication at any time. (**A**) Intake of abortive medication led to significantly higher reduction in pain intensites on the NRS compared to no intake (n = 10; *p* = 0.019). (**B**, **C**) After intake of abortive medication, tear fluid CGRP fell significantly more compared to patients who didn’t take abortive medication using the Abbexa® ELISA (*p* = 0.014, B) or the Cusabio® ELISA (*p* = 0.033, C). Bars represent standard error. AM, abortive medication. * p < 0.05

Ten patients (58.8%) took attack abortive medication (AM) to treat the GTN-induced headache (NSAIDs: n = 7, triptans: n = 3, metamizole: n = 2) after 395 ± 74 min. These patients showed a significantly larger reduction in headache intensity from the ‘headache’ to the ‘post headache’ phase compared to patients without intake of acute medication (NRS: AM: −3.9 ± 1.3; no AM: −2.6 ± 0.7, Mann–Whitney-U-Test: U = 58.500; *p* = 0.019, Fig. 5A). In addition, patients having taken abortive medication showed a reduction of tear fluid CGRP levels from the ‘headache’ to the ‘post headache’ phase (Abbexa® CGRP ELISA: -0.82 ± 1.26 ng/ml, median (IQR) −0.54 (1.28) ng/ml) while patients without intake of abortive medication showed a further increase (+ 1.24 ± 2.06 ng/ml, median (IQR) + 0.41 (2.27) ng/ml; Mann–Whitney-U-Test: U = 60.000, *p* = 0.014, Fig. 5B). Similarly, there was a significantly higher reduction of tear fluid CGRP levels in patients having taken abortive medication compared to patients without medication from the ‘headache’ to the ‘post headache’ phase using the Cusabio® CGRP ELISA (Cusabio® CGRP ELISA: AM: -0.66 ± 1.27 ng/ml, median (IQR)−0.65 (0.9) ng/ml; no AM: −0.12 ± 0.24 ng/ml, median (IQR) −0.15 (0.48) ng/ml, Mann–Whitney-U-Test: U = 57.000, *p* = 0.033, Fig. 5C).

## Discussion

Main finding of this study is that tear fluid CGRP levels rose during GTN-induced experimental headache in migraine patients. Increase of tear fluid CGRP level was more elevated if the headache intensity was strong. Following headache resolution, tear fluid CGRP levels were significantly more reduced in patients having taken abortive medication.

Tear fluid CGRP levels significantly rose in migraine patients developing an experimental delayed headache after GTN application. To the best of our knowledge, this is the first study investigating tear fluid CGRP levels in an experimental migraine attack. These results support our earlier findings, showing elevated tear fluid CGRP levels in ictal migraine patients who had refrained from taking abortive medication compared to interictal patients and healthy controls [12]. To date, few studies investigated CGRP in experimental migraine with inconsistent findings [18, 19]. One study didn’t find any differences in plasma CGRP levels up to 240 min after GTN application in migraine patients developing an experimental headache [19], whereas another study found elevated plasma CGRP levels [18]. In the latter study, plasma CGRP levels were investigated before and 1 h after intravenous GTN application as well as 1 and 2 h after headache onset in antecubital blood of migraine patients and healthy controls. Significantly elevated plasma CGRP levels were found 1 and 2 h after headache onset. Further, headache intensity was positively correlated with CGRP. No headache or CGRP rise was detected in healthy controls. We believe that the detection of tear fluid CGRP is more reliable due to direct trigeminal innervation of the eye, that may lead to higher peptide concentration as well as lower matrix effects in the ELISA.

In our study sample, rise of CGRP levels was significantly higher if headache intensity was strong. As mentioned above, a previous study also found higher CGRP levels dependent of headache intensity [18]. Headache intensities vary between attacks, so differences in increase of CGRP levels or CGRP levels might be one factor contributing to differences in headache attacks. Interestingly, patients with a strong headache intensity reported significantly more headache days/ month and numerically more migraine days/ month. It might be speculated that patients with a higher headache frequency are more sensitive to GTN and develop a more intense headache.

Patients who took abortive medication showed a significantly larger reduction of pain intensity as well as tear fluid CGRP levels compared to patients who refrained from taking acute medication. This is consistent with our previous finding showing unmedicated ictal migraine patients have significantly higher tear fluid CGRP levels compared to patients who took abortive medication [12]. Further, other studies showed a reduction of CGRP levels after triptan use in spontaneous [20, 21] and GTN-induced migraine attacks [17]. CGRP reduction was correlated with the efficacy of triptans since responders to rizatriptan showed a significant reduction of jugular vein CGRP levels starting 1 h and lasting up to 12 h after intake. No change in CGRP levels was detected in non-responders [21]. In patients showing headache improvement of at least 30% after application of sumatriptan 20 mg nasal spray in GTN-induced experimental headache, peripheral blood CGRP levels were significantly reduced 1 h after application [17]. No change in CGRP levels was seen in patients without headache improvement. Taken together, the reduction of plasma CGRP levels is well described after triptan intake. In contrast to earlier studies, most patients in our study cohort used NSAIDs, nonetheless a significant decrease of tear fluid CGRP levels was also observed in this mixed group.

The best method for CGRP measurement in migraine has been a matter of debate. To date, there is no gold-standard method and study findings are heterogenous due to the nature of the peptide itself, its rapid degradation, as well as different detection methods and sampled materials [8, 22–24]. CGRP measurement and evaluation in peripheral blood showed inconsistent findings, likely due to dilution, sampling remote from the cranial release site or degradation by various proteases [8, 22]. Key influencing factors in sampling and assay procedure have been described [8]. In this respect, the detection of CGRP in tear fluid or saliva might be advantageous due to sampling close to the site of release of the peptide and less dilution. Feasibility and validity have been shown in previous studies that detected higher interictal tear fluid and saliva CGRP levels in migraine patients compared to healthy controls, and an increase of saliva CGRP in episodic migraine patients in spontaneous migraine attacks [9–12].

In this study, we used two different CGRP ELISA kits that were both used before for detecting CGRP by other research groups [9, 25]. The choice of detection method is significant for several reasons. CGRP exists in two isoforms, α- and ß-CGRP, that differ in three amino acids. It has been proposed that α-CGRP is more relevant to migraine, while ß-CGRP is more prevalent in gut, but there is a wide overlap. The Abbexa® ELISA was raised against α-CGRP while the Cusabio® ELISA was raised against ß-CGRP due to the manufacturer’s information. However, because of the small difference between the two peptides, it is not clear if current ELISAs are able to reliably differentiate between the two isoforms. Further, it was proposed that the Cusabio® CGRP ELISA might detect the pro-peptide rather than ß-CGRP itself, due to the data sheet as well as negative binding by commercial α- or ß-CGRP [26, 27]. To the best of our knowledge, after presentation of this data at a congress, it hasn’t been published so far.

Results from different ELISAs are not directly comparable due to detection of different epitopes and cross-reactivity between CGRP isoforms [24]. This study shows differences concerning total tear fluid CGRP concentrations showing higher concentrations using the Abbexa® CGRP ELISA compared to the Cusabio® CGRP ELISA. This is a finding that have been made before in peripheral blood and one might speculate that this might lead back to the different binding types [8, 22, 25]. However, much more research in different compartments is needed to further evaluate this assumption.

A conclusion that can be drawn from this study is, that we showed that tear fluid CGRP levels were significantly elevated during a GTN-induced headache, so taken together this data shows that the detection of CGRP in tear fluid is feasible and reliable. The tear fluid CGRP measurement established with the Cusabio® kit in previous studies could be reproduced with two different ELISA kits in the present study. As stated above, absolute values produced by different ELISAs cannot be compared. Relative results were similar between the two ELISAs, however, the Abbexa® kit seemed more sensitive as some differences did not reach significance with the Cusabio® kit. Previous results were reproduced, showing that interictal migraine patients had significantly higher tear fluid CGRP levels compared to healthy controls. There was one difference between the results with the two ELISA kits: with the Abbexa® kit CGRP-levels stayed elevated in the post-headache phase (1–2 h after drug-induced or spontaneous resolution) while the Cusabio® kit detected reduced CGRP levels in this phase. This might be consistent with the Cusabio® kit detecting a pro-peptide rather than CGRP itself (see above), but this interpretation currently remains speculative.

## Limitations

The present study has several limitations. A larger sample size would have enabled the investigation of sub-groups, e.g. migraine patients not developing headache after GTN. Including a healthy control group receiving GTN would have enabled to investigate whether the application of GTN induces CGRP release by itself or only when a delayed headache is induced and/or in migraine patients. Lastly, a cross-over study design with a randomized GTN or placebo application would have further strengthened our results. Further, we could show that tear fluid CGRP levels are elevated during experimental migraine.

## Conclusions

Taken together, detection of CGRP in tear fluid is feasible during acute headache. Our study results support the role of CGRP in GTN-induced experimental headache, similar to what has been shown in spontaneous migraine attacks. This strengthens the use of GTN as a valid human migraine model, especially for the investigation of the role of CGRP in a migraine attack. Further, the results show that tear fluid CGRP is elevated during acute migraine and serve as a biomarker for the migraine attack in future.

## Data Availability

No datasets were generated or analysed during the current study.
